# Systematic Analysis of Mobile Genetic Elements Mediating β-Lactamase Gene Amplification in Noncarbapenemase-Producing Carbapenem-Resistant *Enterobacterales* Bloodstream Infections

**DOI:** 10.1128/msystems.00476-22

**Published:** 2022-08-29

**Authors:** W. C. Shropshire, A. Konovalova, P. McDaneld, M. Gohel, B. Strope, P. Sahasrabhojane, C. N. Tran, D. Greenberg, J. Kim, X. Zhan, S. Aitken, M. Bhatti, T. C. Savidge, T. J. Treangen, B. M. Hanson, C. A. Arias, S. A. Shelburne

**Affiliations:** a Department of Infectious Diseases and Infection Control, The University of Texas MD Anderson Cancer Center, Houston, Texas, USA; b Department of Microbiology and Molecular Genetics, McGovern Medical School, The University of Texas Health Science Center at Houston, Houston, Texas, USA; c Division of Pharmacy, The University of Texas MD Anderson Cancer Center, Houston, Texas, USA; d Department of Internal Medicine, UT Southwestern Medical Center, Dallas, Texas, USA; e Department of Microbiology, UT Southwestern Medical Center, Dallas, Texas, USA; f Department of Bioinformatics, UT Southwestern Medical Center, Dallas, Texas, USA; g Division of Pharmacy, Michigan Medicine at University of Michigan, Ann Arbor, Michigan, USA; h Department of Laboratory Medicine, The University of Texas MD Anderson Cancer Center, Houston, Texas, USA; i Department of Pathology and Immunology, Baylor College of Medicine, Houston, Texas, USA; j Department of Pathology, Texas Children’s Hospital, Houston, Texas, USA; k Department of Computer Science, Rice University, Houston, Texas, USA; l Center for Infectious Diseases, School of Public Health, University of Texas Health Science Center, Houston, Texas, USA; m Department of Medicine, Houston Methodist Hospital, Houston, Texas, USA; n Department of Genomic Medicine, The University of Texas MD Anderson Cancer Center, Houston, Texas, USA; University of Illinois at Chicago

**Keywords:** carbapenem resistance, extended spectrum beta lactamase, mobile genetic elements, multi-drug resistance, osmoporin gene regulation, oxford nanopore technologies

## Abstract

Noncarbapenemase-producing carbapenem-resistant *Enterobacterales* (non-CP-CRE) are increasingly recognized as important contributors to prevalent carbapenem-resistant *Enterobacterales* (CRE) infections. However, there is limited understanding of mechanisms underlying non-CP-CRE causing invasive disease. Long- and short-read whole-genome sequencing was used to elucidate carbapenem nonsusceptibility determinants in *Enterobacterales* bloodstream isolates at MD Anderson Cancer Center in Houston, Texas. We investigated carbapenem nonsusceptible *Enterobacterales* (CNSE) mechanisms (i.e., isolates with carbapenem intermediate resistance phenotypes or greater) through a combination of phylogenetic analysis, antimicrobial resistance gene detection/copy number quantification, porin assessment, and mobile genetic element (MGE) characterization. Most CNSE isolates sequenced were non-CP-CRE (41/79; 51.9%), whereas 25.3% (20/79) were *Enterobacterales* with intermediate susceptibility to carbapenems (CIE), and 22.8% (18/79) were carbapenemase-producing *Enterobacterales* (CPE). Statistically significant copy number variants (CNVs) of extended-spectrum β-lactamase (ESBL) genes (Wilcoxon Test; *P*-value < 0.001) were present in both non-CP-CR E. coli (median CNV = 2.6×; *n* = 17) and K. pneumoniae (median CNV = 3.2×, *n* = 17). All non-CP-CR E. coli and K. pneumoniae had predicted reduced expression of at least one outer membrane porin gene (i.e., *ompC/ompF* or *ompK36/ompK35*). Completely resolved CNSE genomes revealed that IS*26* and IS*Ecp1* structures harboring *bla*_CTX-M_ variants along with other antimicrobial resistance elements were associated with gene amplification, occurring in mostly IncFIB/IncFII plasmid contexts. MGE-mediated β-lactamase gene amplifications resulted in either tandem arrays, primarily mediated by IS*26* translocatable units, or segmental duplication, typically due to IS*Ecp1* transposition units. Non-CP-CRE strains were the most common cause of CRE bacteremia with carbapenem nonsusceptibility driven by concurrent porin loss and MGE-mediated amplification of *bla*_CTX-M_ genes.

**IMPORTANCE** Carbapenem-resistant *Enterobacterales* (CRE) are considered urgent antimicrobial resistance (AMR) threats. The vast majority of CRE research has focused on carbapenemase-producing *Enterobacterales* (CPE) even though noncarbapenemase-producing CRE (non-CP-CRE) comprise 50% or more of isolates in some surveillance studies. Thus, carbapenem resistance mechanisms in non-CP-CRE remain poorly characterized. To address this problem, we applied a combination of short- and long-read sequencing technologies to a cohort of CRE bacteremia isolates and used these data to unravel complex mobile genetic element structures mediating β-lactamase gene amplification. By generating complete genomes of 65 carbapenem nonsusceptible *Enterobacterales* (CNSE) covering a genetically diverse array of isolates, our findings both generate novel insights into how non-CP-CRE overcome carbapenem treatments and provide researchers scaffolds for characterization of their own non-CP-CRE isolates. Improved recognition of mechanisms driving development of non-CP-CRE could assist with design and implementation of future strategies to mitigate the impact of these increasingly recognized AMR pathogens.

## INTRODUCTION

Carbapenem-resistant *Enterobacterales* (CRE) infections are major public health challenges, particularly within vulnerable patient populations (1–6). There is a strong association between carbapenem resistance and resistance to other antibiotics (multidrug resistance; MDR), in part because carbapenem-resistant infections commonly occur in patients who have previously received multiple courses of antimicrobials (7, 8). A primary factor responsible for the dissemination of MDR phenotypes are mobile genetic elements (MGEs). These complex genetic structures (e.g., plasmids, transposons, and integrons) can mobilize carbapenem resistance determinants in addition to other antimicrobial resistance (AMR) genes that confer resistance to other classes of antibiotics such as fluoroquinolones, aminoglycosides, and other novel β-lactam/β-lactamase inhibitor combinations (9–13). In recent years, the development of long-read sequencing technologies has improved our understanding of the complexity, diversity, and prevalence of these MGEs as key drivers of MDR infections (13–20).

There are two general mechanisms by which MGEs contribute to the development of carbapenem resistance in *Enterobacterales* (21). MGEs can disseminate and mobilize carbapenemase genes, which encode enzymes that are able to hydrolyze the carbapenem β-lactam ring with sufficient efficiency to inactivate the drug, through horizontal gene transfer pathways (11, 22). For example, there are well documented associations of the Klebsiella pneumoniae carbapenemase (KPC) encoding gene being disseminated through isoforms of the Tn*3*-based Tn*4401* transposon (23). Interestingly, in recent years, surveillance studies have found that up to 50% of CRE detected lack a carbapenemase gene, i.e., are noncarbapenemase-producing CRE (non-CP-CRE) (1–3). Similar to MGEs key role in dissemination of carbapenemases, MGEs are also necessary for the dissemination of extended-spectrum β-lactamase (ESBL) and AmpC-like encoding enzymes that are both critical for the development of the non-CP-CRE phenotype (11, 12, 24–28).

Much of the existing knowledge regarding non-CP-CRE mechanisms is derived from laboratory passaging or serial, single isolate studies (24–28). These studies have shown that non-CP-CRE development typically involves increased expression or gene copy number of ESBL or AmpC-like enzymes in conjunction with outer membrane porin (*omp*) gene inactivation, which results in a reduced carbapenem concentration in the periplasmic space (24–28). Given that both ESBL and AmpC-like encoding genes are typically located in MGEs (11, 13, 29), an increase in β-lactamase gene copy number would seem to be feasible for a broad array of ESBL and AmpC-like positive *Enterobacterales*.

Recent data indicate that both non-CP-CRE and carbapenemase-producing *Enterobacterales* (CPE) undergo multiple genomic and transcriptomic adaptations prior to becoming fully resistant to carbapenems (30, 31). A CRE US-based surveillance study published in 2020 found a large proportion of “unconfirmed” CRE infections (1) with clinical outcomes comparable to confirmed CRE infections, suggesting that many CRE isolates may have unstable, borderline carbapenem resistance (i.e., carbapenem intermediate resistance). Considering that this instability of carbapenem resistance phenotype may be due to heteroresistance arising from gene amplifications (32), it is critical to better understand the full breadth of carbapenem resistance genotypes. Therefore, one aim of this study is to characterize the union of *Enterobacterales* bloodstream isolates that are carbapenem-intermediate or carbapenem-resistant, hereinto referred to as carbapenem nonsusceptible *Enterobacterales* (CNSE), that contribute to carbapenem resistance in the hospital setting.

While many studies have shown associations of β-lactamase gene copy numbers with increased β-lactam phenotype (13–17, 33), to our knowledge, a systematic analysis of MGE-mediated β-lactamase-encoding gene amplifications in a large cohort of CNSE isolates using completed genome assemblies has not been performed. Given the repetitive, complex nature of MGEs that harbor these β-lactamase encoding genes, PCR detection or short-read sequencing approaches have had limited capacity to reveal the breadth of MGEs contributing to these varied CRE phenotypes.

Herein, we sought to systematically determine carbapenem resistance mechanisms by applying a combination of short- and long-read sequencing to a well-defined cohort of CNSE isolates. We found that non-CP-CRE isolates caused the vast majority of our CRE bacteremia cases and harbored MGEs with complex arrangements primarily of ESBLs, such as *bla*_CTX-M_ variants, mediated by either IS*26* or IS*Ecp1* elements. There was a statistically significant association of ESBL amplification in conjunction with *omp* gene disruption in non-CP-CR Escherichia coli and Klebsiella pneumoniae. Using Oxford Nanopore Technologies (ONT) long-read sequencing, we clarified that ESBL amplification was associated with IS*26*-mediated “translocatable units” (TUs) and IS*Ecp1* “transposition units” (TPUs) in both non-CP-CR Escherichia coli and Klebsiella pneumoniae, thereby improving the understanding of mechanisms underlying the non-CP-CRE phenotype.

## RESULTS

### Molecular epidemiology of carbapenem-nonsusceptible *Enterobacterales* (CNSE) causing bacteremia at MD Anderson Cancer Center (MDACC).

There were 1,632 unique *Enterobacterales* bloodstream infections (BSIs) at our institution from July 2016 to June 2020. The leading causes were Escherichia coli (939/1,632; 57.5%) followed by Klebsiella pneumoniae (338/1,632; 20.7%) and Enterobacter spp. (159/1,632; 9.7%). A total of 5.2% (85/1,632) were CDC-defined carbapenem-resistant with an additional 1.8% (29/1,632) having intermediate carbapenem resistance based on CLSI breakpoints (i.e., carbapenem-intermediate *Enterobacterales* [CIE]), resulting in a total 7.0% (114/1,632) that were carbapenem-nonsusceptible *Enterobacterales* (CNSE) as initially determined by the MDACC clinical microbiology laboratory. When stratifying the causal species of BSI by carbapenem nonsusceptibility, 39.5% (45/114) of CNSE were Escherichia coli followed by Klebsiella pneumoniae *sensu stricto* (30.7%; 35/114) and Enterobacter spp. (16.7%; 19/114). We found a statistically significant difference in carbapenem nonsusceptibility by species (Fisher’s exact test, *P* value < 0.001) with a higher prevalence of K. pneumoniae BSIs (10.4%; 35/338) that were carbapenem-nonsusceptible compared to E. coli (4.8%; 45/939), consistent with other CRE surveillance studies in the United States (1, 2, 34).

**FIG 1:**
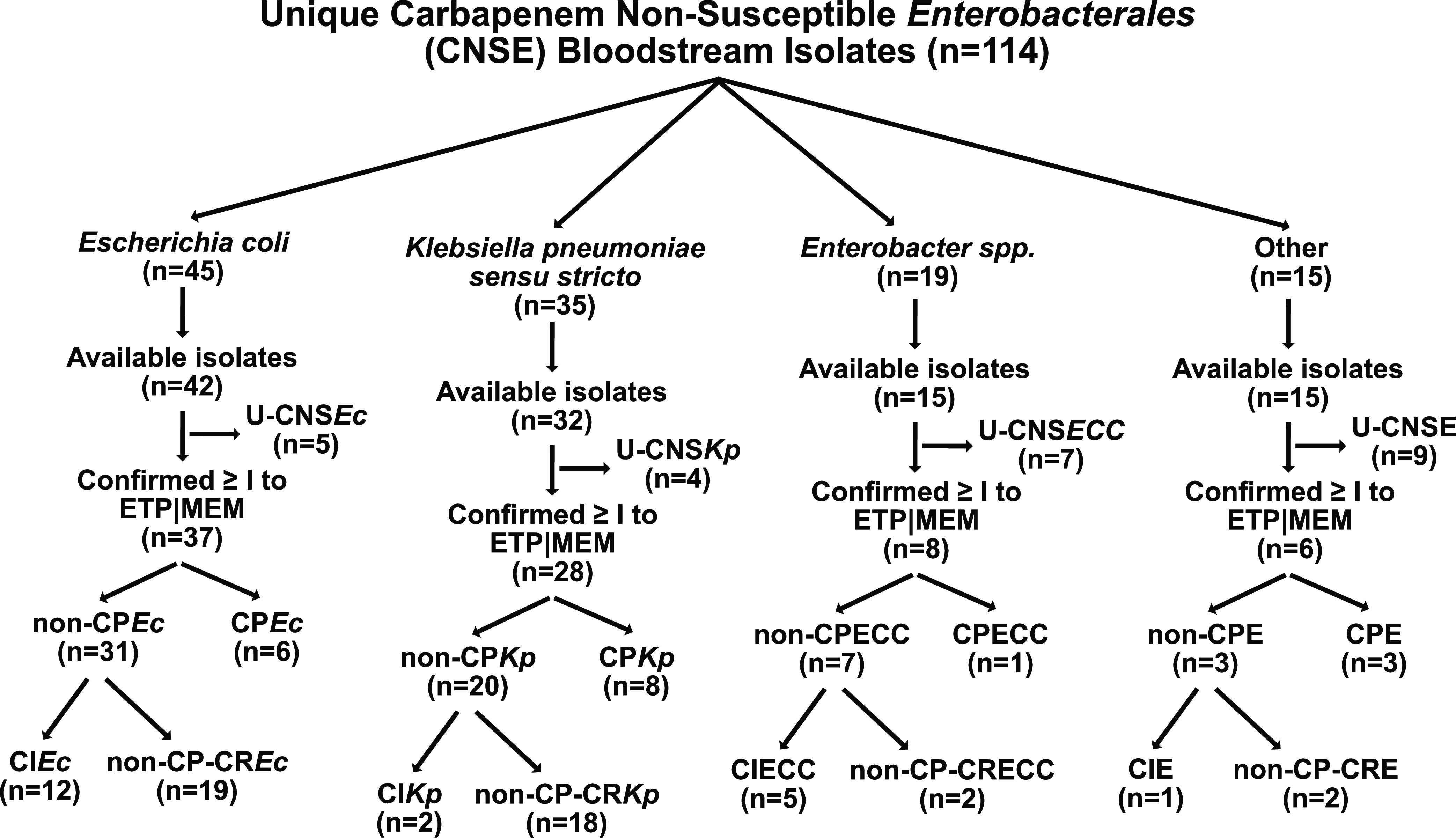
Selection and delineation of carbapenem-nonsusceptible *Enterobacterales* bloodstream infection isolates. Total isolates per group included in parenthesis. U-CNS, unconfirmed carbapenem-nonsusceptible; non-CP, noncarbapenemase-producing; non-CP-CR, noncarbapenemase-producing carbapenem-resistant; CP, carbapenemase producing; *Ec*, Escherichia coli; *Kp*, Klebsiella pneumoniae; ECC, Enterobacter cloacae complex; E, *Enterobacterales*.

A total of 91% (104/114) CNSE BSI isolates were present in our sample collection (Fig. 1). Of these 104 CNSE BSI isolates, we confirmed at least ertapenem MIC intermediate interpretations for 37/42 E. coli (88%), 28/32 K. pneumoniae (88%), 8/15 Enterobacter spp. (53%), and 6/15 other *Enterobacterales* (40%), with the remaining isolates being considered unconfirmed-CNSE (Fig. 1). Thus, we had 79 CNSE-confirmed BSI isolates which underwent whole-genome sequencing (WGS) to determine respective carbapenem nonsusceptibility genotypes. Only 23% of BSI isolates (18/79) had a confirmed carbapenemase, whereas the majority were non-CP-CRE (41/79; 52%) or CIE (20/79; 25%) based on WGS analysis and carbapenem MIC determination (Fig. 1). We identified 17 CNS*Ec* bacteremia cases that had a prior initial carbapenem-susceptible E. coli bacteremia infection which had tested positive for ESBL production in 16/17 cases. Interestingly, all 17 of these CNS*Ec* isolates were carbapenemase-negative. Similarly, 5/6 CNS*Kp* that were preceded by an initial carbapenem-susceptible K. pneumoniae bacteremia were carbapenemase-negative as well. When focusing on clinical features, there were no statistically significant differences in age, gender, country of origin, recent travel history, or predicted source of BSI across each of the CNSE categories, albeit there were a small number of observations per category (Table S1).

10.1128/msystems.00476-22.1 TABLE S1 Demographic and clinical epidemiology of CNSE blood stream infections July 2016 to June 2020 at MDACC. Download Table S1, XLSX file, 0.01 MB. Copyright © 2022 Shropshire et al. 2022 Shropshire et al. https://creativecommons.org/licenses/by/4.0/ This content is distributed under the terms of the Creative Commons Attribution 4.0 International license.

Enterobacter spp. were the third most prevalent group of CNSE BSI isolates with all isolates belonging to the Enterobacter cloacae complex (ECC) (Table S2). The majority of CNSE-confirmed ECC had CIE phenotypes (5/8; 63%), with only one carbapenemase-producing ECC (CPECC) isolate harboring *bla*_KPC-2_ (MB8139), and two noncarbapenemase-producing carbapenem-resistant ECC (non-CP-CRECC). With regard to the non-CP-CRECC isolates, both had outer membrane porin (*omp*) gene disruptions with one non-CP-CRECC (MB5921) containing an ESBL gene (*bla*_SHV-12_). The other non-CP-CRECC isolate (MB6956) had a carbapenem-resistant mechanism that likely involved an overexpressed chromosomal *ampC* gene (*bla*_CMH_) due to an *ampD*/*ampE* fusion mutation, with the inactivation of the AmpD gene predicted to result in AmpC derepression (35) (Table S2). The six other *Enterobacterales* spp. detected in our cohort included 3 CPE (Klebsiella spp. not including K. pneumoniae *sensu stricto*), 2 non-CP-CRE (1 K. aerogenes and 1 Citrobacter freundii), and 1 CIE (Serratia marcescens) (Table S2). We focused the remainder of this study on the two most common, clinically relevant species in our cohort, E. coli and K. pneumoniae, and the putative mechanisms responsible for their carbapenem-nonsusceptible phenotypes.

10.1128/msystems.00476-22.2 TABLE S2 Short read sequencing data by individual isolate. Download Table S2, XLSX file, 0.02 MB. Copyright © 2022 Shropshire et al. 2022 Shropshire et al. https://creativecommons.org/licenses/by/4.0/ This content is distributed under the terms of the Creative Commons Attribution 4.0 International license.

### Characterization of carbapenem resistance mechanisms among CNS E. coli and K. pneumoniae isolates.

**FIG 2:**
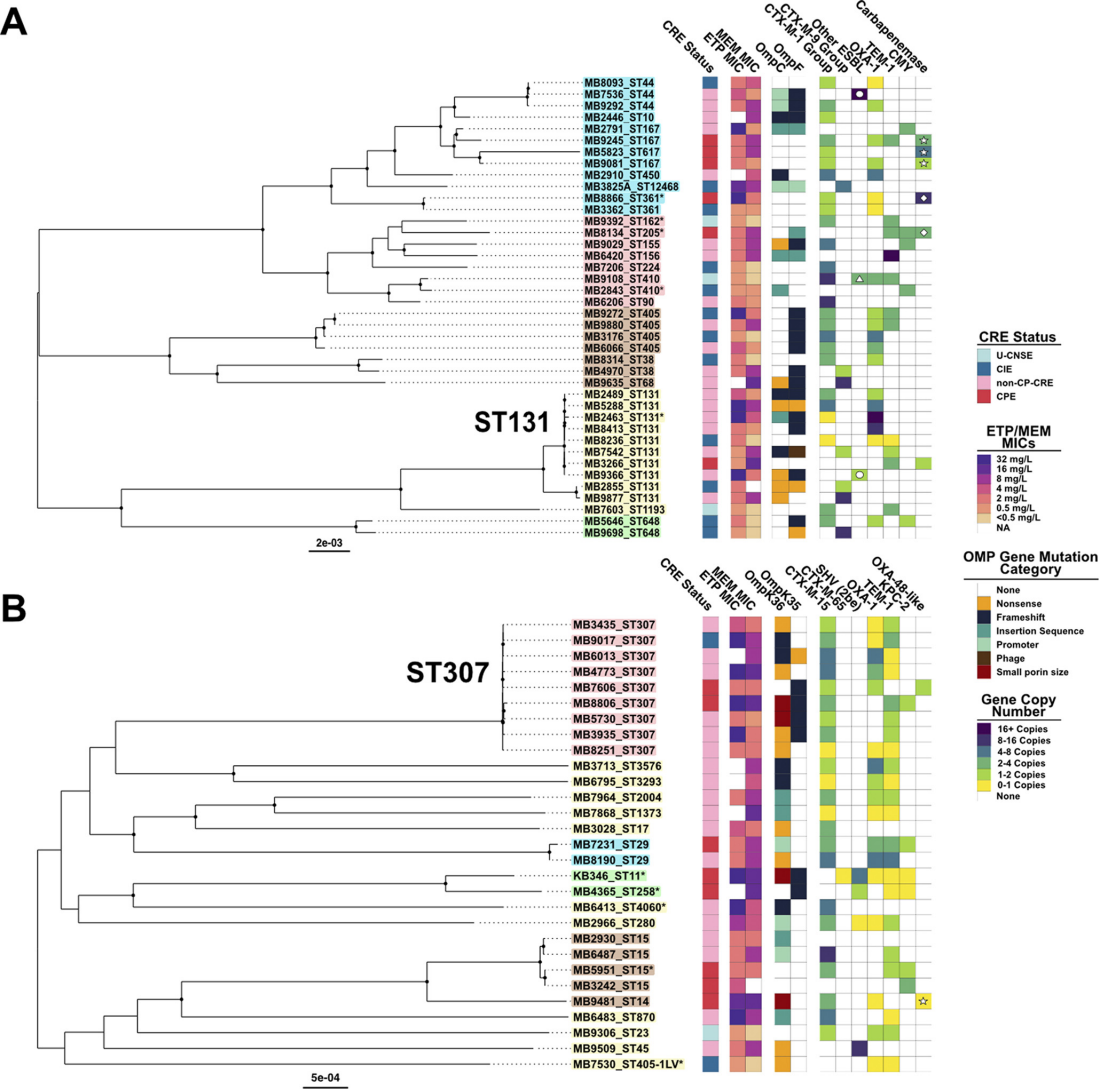
Population structure of E. coli and K. pneumoniae bacteremia isolates with phenotype/genotype data. Core gene alignment inferred; midpoint rooted maximum likelihood phylogenies. Circles at internal nodes indicate UFBoot values with ≥95% support. Tip label background color corresponds to nested population structure identified using hierarchical clustering of sequence data with rhierbaps. Carbapenem resistance status, ertapenem (ETP) and meropenem (MEM) MICs (μg/mL), outer membrane porin gene mutation status, and gene copy number estimate are presented in columnar data from left to right and labeled in the legend, respectively. An asterisk (*) adjacent to the tip label indicates isolates with only draft assembly. Samples with ETP or MEM MIC results labeled “NA” indicate isolates that did not have these data recorded by the MDACC clinical microbiology lab. (A) E. coli population structure (*n* = 40). Circles in the “Other ESBL” column indicate *bla*_TEM_ variants whereas the triangle indicates *bla*_SHV-12_. Stars in the “Carbapenemase” column indicate *bla*_NDM-5_, diamonds indicate *bla*_OXA-48-like_, and absence of shape indicates *bla*_KPC-2_. Tip labels correspond to hierarchical population structure cluster by phylogroup with A (*n* = 12; blue), B2 (*n* = 11; yellow), D (*n* = 7; brown), B1/C (*n* = 8; pink), and F (*n* = 2; green) (B) K. pneumoniae population structure (*n* = 29). The isolate with a star in the “OXA-48-like” (*bla*_OXA-48-like_) column indicates cocarriage of *bla*_NDM-1_ with 1 to 2 copies.

There were 37 unique carbapenem-nonsusceptible E. coli (CNS*Ec*) bacteremia isolates with 6 CP*Ec* (16%), 19 non-CP-CR*Ec* (51%), and 12 CI*Ec* (32%) (Table S2). A summary of molecular features of CNS*Ec* is provided in Table S3. Core gene alignment inferred, maximum-likelihood phylogenetic trees for CNS*Ec* isolates with carbapenem susceptibility profile, outer membrane porin gene (*omp*) mutation status, and β-lactamase gene presence/absence with copy number estimates are shown in Fig. 2A. Hierarchical clustering of core gene SNPs resulted in five clusters, indicated by tip label color (Fig. 2A), that segregate isolates based on phylogroups A (*n* = 12), B2 (*n* = 11), D (*n* = 7), B1/C (*n* = 8), and F (*n* = 2) (36). The most identified sequence type (ST) among CNS*Ec* was the uropathogenic strain ST131 (10/37; 27%). The mean pairwise core gene SNP difference was 57,355 SNPs (standard deviation [SD] = 25,621 SNPs). There were only two clinical isolates, MB9272 and MB9880, that had less than 50 core gene SNP differences (18 SNPs), further indicating minimal clonal infections among the E. coli strains in our cohort.

10.1128/msystems.00476-22.3 TABLE S3 Molecular epidemiology of E. coli bloodstream infections July 2016 to June 2020 at MDACC. Download Table S3, XLSX file, 0.01 MB. Copyright © 2022 Shropshire et al. 2022 Shropshire et al. https://creativecommons.org/licenses/by/4.0/ This content is distributed under the terms of the Creative Commons Attribution 4.0 International license.

Among the six CP*Ec* isolates, three isolates from phylogroup A harbored *bla*_NDM-5_, two unique ST isolates harbored plasmid borne *bla*_OXA-48-like_ genes (MB8866 = *bla*_OXA-232_ and MB8134 = *bla*_OXA-181_), and one isolate (MB3266) carried a plasmid-borne Tn*4401a* transposon harboring *bla*_KPC-2_. Only one CP*Ec* (MB8134) had an *omp* mutation (IS*2* insertion within *ompF*) (Fig. 2A). Regarding non-CP-CR*Ec*, 79% (15/19) of isolates were ESBL-positive. The most common β-lactamases detected in non-CP-CR*Ec* were CTX-M-1 group variants (7 *bla*_CTX-M-15_ and 3 *bla*_CTX-M-55_), CTX-M-9 group variants (3 *bla*_CTX-M-27_, 1 *bla*_CTX-M-14_, and 1 *bla*_CTX-M-195_), *bla*_OXA-1_ (*n* = 8), *bla*_TEM-1_ (*n* = 4), and *bla*_CMY_ variants (*n* = 2). One ST131 non-CP-CR*Ec* isolate (MB9366) carried a novel *bla*_TEM_ variant (p.M182T, p.G238S, p.E240K, p.S243A, p.S270G), which was identified as an ESBL-E by the MDACC clinical microbiology lab and has an antibiogram that resembles an ESBL-E (Table S4). In contrast to the low prevalence of *ompC* and *ompF* mutations detected in CP*Ec*, all 19 non-CP-CR*Ec* isolates had at least one *ompC* or *ompF* mutation except for MB6206 (Fig. 2A; Table S3), which had an IS*Ecp1*-*bla*_CTX-M-55_ insertion into the histidine kinase gene *envZ*, a known regulator of *ompC* and *ompF* expression (37). Consistent with EnvZ inactivation, immunoblot analysis confirmed a significant reduction of OmpC/OmpF in MB6206 (Fig. S1). Furthermore, 63% (12/19) of non-CP-CR*Ec* isolates were double mutant *ompC/ompF* isolates (Fig. 2A; Table S3). Similar to non-CP-CR*Ec*, 11/12 (91.7%) of CI*Ec* were ESBL carriers with eight CTX-M-1 group variants (7 *bla*_CTX-M-15_; 1 *bla*_CTX-M-1_) and three CTX-M-9 group variants (2 *bla*_CTX-M-14_; 1 *bla*_CTX-M-27_). Other common β-lactamases detected in CI*Ec* were *bla*_OXA-1_ (*n* = 7) and *bla*_CMY_ (*n* = 2) variants. Relative to non-CP-CR*Ec* (18/19), CI*Ec ompC* and *ompF* mutations were less prevalent (7/12; 58%; Fisher’s exact test *P* value = 0.02) with only two strains (16%) having mutations in both genes.

10.1128/msystems.00476-22.4 TABLE S4 Antimicrobial susceptibility data by individual isolate. Download Table S4, XLSX file, 0.02 MB. Copyright © 2022 Shropshire et al. 2022 Shropshire et al. https://creativecommons.org/licenses/by/4.0/ This content is distributed under the terms of the Creative Commons Attribution 4.0 International license.

10.1128/msystems.00476-22.7 FIG S1 Immunoblot analysis of OmpC/OmpF protein levels. Isolate MB6206 (red box) with IS*Ecp1*-*bla*_CTX-M-55_ insertion into the *envZ* gene displays reduced OmpC/OmpF levels as compared to clinical and lab positive control (MB3266 and K-12 WT, respectively). A series of clinical (MB2489, 3825a, 3362) and K-12 derived *omp* gene knockouts were used to aid with the band identification. *Note: MB3266, 2489, and 6206 contain a 4-amino acid insertion in OmpA protein as compared to K-12, resulting in a change of migration pattern. Immunoblot analysis has been previously described (Tata M, Konovalova A. mBio 10:e00660-19, 2019, https://doi.org/10.1128/mBio.00660-19). Briefly, cell lysates were prepared from exponential phase cultures, grown in lysogenic broth. Samples were normalized by optical density at 600 nm (OD_600_). Proteins were separated on SDS polyacrylamide gels supplemented with 4 molar urea. Immunoblots were developed with previously validated polyclonal rabbit antibodies raised against OmpA, OmpC, and OmpF (Misra R, Reeves P. J Bacteriol 169:4722–4730, 1987, https://doi.org/10.1128/jb.169.10.4722-4730.1987; and Zimmermann R, Wickner W. J Biol Chem 258:3920–3925, 1983, https://doi.org/10.1016/S0021-9258(18)32755-8), and visualized using the ChemiDoc MP Imaging System (Bio-Rad). Download FIG S1, EPS file, 0.1 MB. Copyright © 2022 Shropshire et al. 2022 Shropshire et al. https://creativecommons.org/licenses/by/4.0/ This content is distributed under the terms of the Creative Commons Attribution 4.0 International license.

There were 28 unique carbapenem-nonsusceptible K. pneumoniae (CNS*Kp)* bacteremia isolates with eight CP*Kp* (29%), 18 non-CP-CR*Kp* (64%), and two CI*Kp* (7%) (Table S2). The core population structure of CNS*Kp* BSI isolates is presented in Fig. 2B The finding that 64% CR*Kp* were noncarbapenemase producers was noteworthy given that in most US-based CRE surveillance studies, the majority of CR*Kp* are carbapenemase-positive (1, 34). Indeed, for our cohort, the proportion of non-CP-CR*Kp* (18/28) was comparable to non-CP-CR*Ec* isolates (19/37; χ-squared test statistic = 0.62; *P*-value = 0.4). The most common sequence type identified was the ST307 lineage (9/28; 32%) followed by 18% (5/28) belonging to clonal group 15 (CG15). Hierarchical clustering demonstrated that, apart from ST307 and CG15 isolates, most CNS*Kp* belonged to single, long-branching isolates (Fig. 2B), indicating limited genetic relatedness. In support of this observation was a mean pairwise core gene SNP difference of 22,141 SNPs (SD = 7,864) with the minimum pairwise core gene SNP distance between our CNS*Kp* isolates being 38 SNPs between two ST15 isolates (MB5951 and MB3242). Among CP*Kp*, six isolates encoded *bla*_KPC-2_, one isolate (MB7606) encoded *bla*_OXA-181_, and one isolate (MB9481) encoded two carbapenemases, *bla*_NDM-1_ and *bla*_OXA-48_. The *ompK36* or *ompK35* mutations (i.e., *ompC* and *ompF* K. pneumoniae homologs, respectively) that would be predicted to affect outer membrane porin function were present in 5/8 (62.5%) CP*Kp*. Almost all non-CP-CR*Kp* carried *bla*_CTX-M-15_ (16/18; 84%) with one such isolate having a novel, single amino acid *bla*_CTX-M-15_ variant (MB6013; p.P269S). The β-lactamase-encoding genes *bla*_OXA-1_ (*n* = 14) and *bla*_TEM-1_ (*n* = 10) were also commonly detected in non-CP-CR*Kp.* All non-CP-CR*Kp* isolates had an *ompK36* mutation with 16.6% (3/18) also having an *ompK35* mutation (Fig. 2B). Only 2 CI*Kp* isolates were identified, both having *ompK36* disrupted ORFs with one isolate (MB9017) harboring *bla*_CTX-M-15_, *bla*_OXA-1_, and *bla*_TEM-1,_ and the other isolate harboring only *bla*_OXA-1_ and *bla*_TEM-1._ Taken together, the core population structure indicates disparate CNS E. coli and K. pneumoniae sequence types with little evidence of clonal outbreaks in addition to a high prevalence of ESBL-encoding genes with universal predicted *omp* gene disruption within non-CP-CRE isolates.

### Copy number variant profiling of β-lactamase-encoding genes in CNSE.

**FIG 3:**
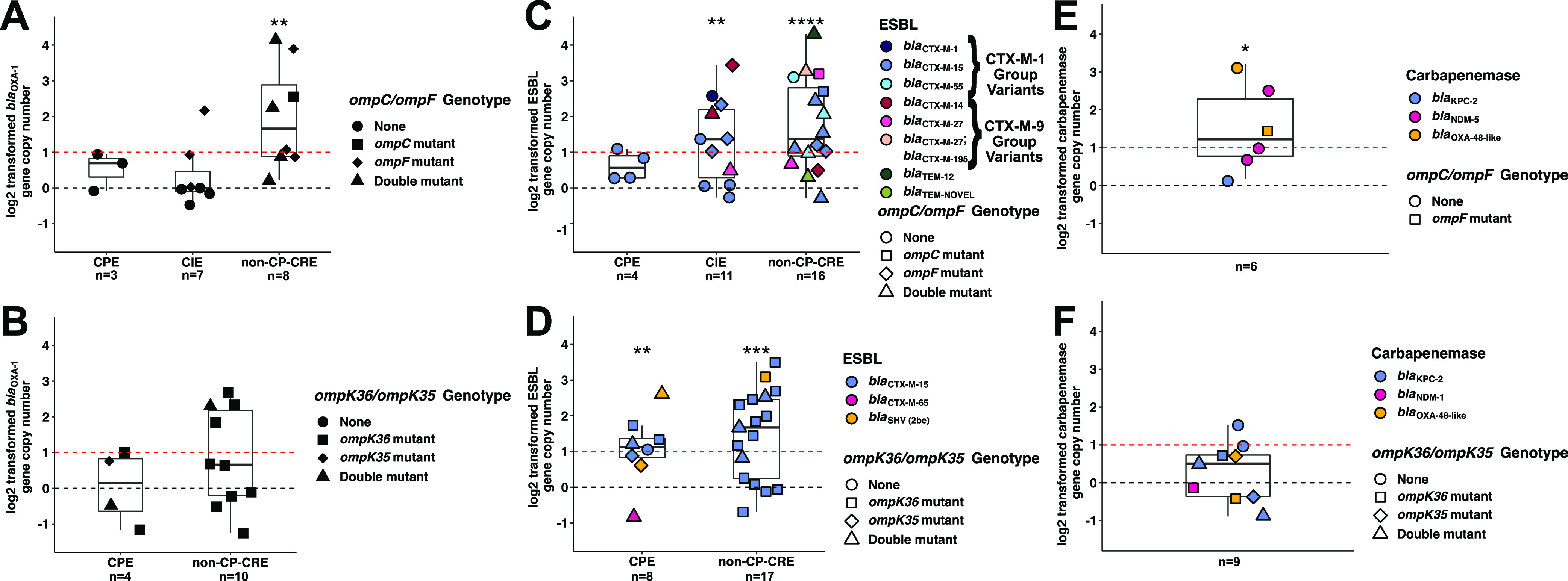
Log_2_ transformed β-lactamase gene copy numbers with outer membrane porin gene mutation profile stratified by carbapenem-nonsusceptible (CNS) definitions. (A, C, and E) Escherichia coli and (B, D, and F) Klebsiella pneumoniae CNS isolates. Black dotted horizontal line at y = 0 is equivalent to 1× gene copy; Red dotted horizontal line at y = 1 is equivalent to 2× gene copy. Totals below categories reflect gene counts. CPE, carbapenemase-producing *Enterobacterales*; CIE, carbapenem-intermediate *Enterobacterales*; non-CP-CRE, noncarbapenemase-producing carbapenem-resistant *Enterobacterales.* One sample, one-sided, Wilcoxon test on nontransformed copy number estimates to determine statistically significant gene copy number amplifications (i.e., >1 copy) with *P*-values: *, *P* < 0.05; **, *P* < 0.01; ***, *P* < 0.001; ****, *P* < 0.0001.

An increase in copy number of ESBL, AmpC-like, and narrow-spectrum β-lactamase-encoding genes has been previously documented as contributing to CNSE development (13, 25, 27, 28). Thus, we next sought to comprehensively assess the presence of β-lactamase gene amplifications and their associations with each carbapenem nonsusceptibility profile (Table S5). To this end, we analyzed β-lactamase-encoding gene copy number variants (CNVs) and determined which CNSE groups had median CNV estimates greater than baseline (i.e., 1 copy) (Fig. 3). Non-CP-CR*Ec* contained statistically significant increases in gene copy numbers of the narrow spectrum β-lactamase-encoding gene *bla*_OXA-1_ (median CNV = 3.4×; one-sample, one-sided, Wilcoxon test *P*-value = 0.004) (Fig. 3A) that were not found in other CNS*Ec* categories nor in any of the CNS*Kp* groups (Fig. 3B). Both non-CP-CR*Ec* (median CNV = 2.6×; Wilcoxon test *P*-value <0.0001) and non-CP-CR*Kp* (median CNV = 3.2×; Wilcoxon test *P*-value <0.001) had statistically significant increases in ESBL gene copy numbers shown in Fig. 3C and D, respectively. Notably 80% (12/15) and 64% (11/17) of ESBL-positive, non-CP-CR*Ec* and non-CP-CR*Kp*, respectively, had an estimated ≥2 copies of ESBL-encoding genes (Table S5). Similar to non-CP-CR*Ec*, CI*Ec* also had a statistically significant increase in ESBL gene copy number (median CNV = 2.6×; *P*-value <0.001). Amplification of carbapenemase-encoding genes (median CNV = 2.4×; *P*-value = 0.02) was also detected in CP*Ec* (Fig. 3E), which was not evident in CP*Kp* (median CNV = 1.4×; *P*-value = 0.2) (Fig. 3F). While there was notably high *bla*_TEM-1b_ amplification in non-CP-CR*Ec* (median CNV = 11.5×), this did not reach statistical significance likely due to small number of observations (*n* = 4) and high variance in CNV estimates (Fig. S2A); whereas non-CP-CR*Kp* did not have evidence of *bla*_TEM-1b_ amplification (Fig. S2B). Lastly, *bla*_CMY_ amplification was present in CNS*Ec* with all five *bla*_CMY_-positive isolates having estimated copy numbers greater than two (Table S5). Thus, a broad range of β-lactamases had evidence of gene copy number amplifications with statistically significant ESBL gene amplifications being detected in both non-CP-CR*Ec* and non-CP-CR*Kp* isolates.

10.1128/msystems.00476-22.5 TABLE S5 Gene amplification data. Download Table S5, XLSX file, 0.01 MB. Copyright © 2022 Shropshire et al. 2022 Shropshire et al. https://creativecommons.org/licenses/by/4.0/ This content is distributed under the terms of the Creative Commons Attribution 4.0 International license.

10.1128/msystems.00476-22.8 FIG S2 *bla*_TEM-1_ log2 transformed copy number estimates for (A) E. coli and (B) K. pneumoniae stratified by CNSE group. Black dotted horizontal line at y = 0 is equivalent to 1× gene copy; red dotted horizontal line at y = 1 is equivalent to 2× gene copy. Download FIG S2, EPS file, 0.2 MB. Copyright © 2022 Shropshire et al. 2022 Shropshire et al. https://creativecommons.org/licenses/by/4.0/ This content is distributed under the terms of the Creative Commons Attribution 4.0 International license.

### Genomic structures contributing to carbapenem resistance development in CNSE cohort.

Having quantified the extent of β-lactamase amplification across each of the CNSE groups, we used long-read ONT sequencing to complete genomes of 65 CNSE isolates (Table S2) in order to resolve the putative MGEs associated with mobilization and amplification of β-lactamase-encoding genes. We initially characterized the MGEs in CNSE isolates harboring β-lactamase genes greater than or equal to 2× copies (Fig. 3) with results shown for CNS*Ec* (Table 1) and CNS*Kp* (Table 2). When we subset these isolates with complete genomes available, we found the majority of CNS*Ec* (21/27; 78%) and CNS*Kp* (12/15; 80%) had MGE *in situ* tandem or *ex situ* segmental duplication associated with the increased β-lactamase copy numbers (Table 1 and 2, respectively). Furthermore, with rare exception, these β-lactamase amplifications were associated with observed insertion sequences IS*26* and/or IS*Ecp1* within the CNSE genomes (Table 1 and 2). Stratifying by species and using nomenclature established for these aforementioned MGEs (29), for the 21 CNS*Ec* with MGE mediated β-lactamase gene amplification, 11 (52%) had IS*26* TUs, 8 (38%) had IS*Ecp1* TPUs, and one isolate had both mechanisms (Table 1). Conversely, of the 12 CNS*Kp* with at least two copies of β-lactamase-encoding genes driven by MGEs, eight (67%) had TPUs, three had (25%) TUs, and one isolate had both mechanisms (Table 2). Thus, IS*26*-mediated TU or IS*Ecp1*-mediated TPU amplifications were primarily associated with MGE inter- and intramolecular mobilization of β-lactamases that contributed to carbapenem nonsusceptibility.

**TABLE 1:** Summary of carbapenem nonsusceptibility mechanisms for E. coli with β-lactamase amplifications^*a*^

Carbapenem resistance status	Sample	MLST	*ompC* ^*b*^	*ompF* ^*b*^	*envZ/ompR* ^*b*^	ESBL CNV	OXA-1 CNV	CMY CNV	TEM CNV	CARB CNV	MGE + β-lactamase^*c*^	Genomic context	Amp. pred.^*d*^
CI*Ec*	MB8314	38	ND	ND	ND	2.6	1.0	ND	ND	ND	IS*Ecp1*-*bla*_CTX-M-15_/*bla*_OXA-1_;IS*Ecp1-bla*_CTX-M-15_	Chromosome	MGE
	MB7206	224	ND	ND	ND	6.0	ND	ND	ND	ND	IS*26*-*bla*_CTX-M-1_	Chromosome	MGE
	MB3176	405	ND	p.Y254EfsX3	ND	5.0	4.5	ND	ND	ND	IS*26*-v1-*bla*_CTX-M-15_/*bla*_OXA-1_	Plasmid	MGE
	MB9272	405	ND	p.Y254EfsX3	ND	2.0	1.9	ND	2.0	ND	IS*26*-*bla*_CTX-M-15_/*bla*_OXA-1_/*bla*_TEM-1b_	Plasmid	PCN
	MB2843	410	Insertion sequence	ND	ND	ND	ND	4.0	ND	ND	IS*Ecp1*-*bla*_CMY-2_	Chromosome	MGE
	MB5646	648	ND	p.A238GfsX7	ND	2.6	1.0	1.9	ND	ND	IS*Ecp1*-*bla*_CTX-M-15_;IS*26*-*bla*_OXA-1_;*bla*_CMY-4_	Plasmid	PCN
	MB9698	648	ND	p.L15X	ND	10.8	ND	ND	ND	ND	IS*Ecp1*-*bla*_CTX-M-14_	Chromosome	MGE
	MB3825A	12468	IS*1A* 50 bp +1 ATG	IS*1A* 101 bp +1 ATG	ND	4.2	ND	ND	ND	ND	IS*Ecp1*-*bla*_CTX-M-14_	Chromosome + plasmid	MGE
Non-CP-CR*Ec*	MB2446	10	p.F212RfsX30	p.Y26WfsX9	ND	2.0	ND	ND	ND	ND	IS*Ecp1*-*bla*_CTX-M-55_	Plasmid	MGE + PCN*
	MB7536	44	IS*1F* 66 bp +1 ATG	p.Y62YfsX1	ND	19.8	ND	ND	19.0	ND	IS*26*-*bla*_TEM-12_(Tn3-like transposon)**	Plasmid	MGE + PCN
	MB9292	44	IS*1R* 49 bp +1 ATG	p.S46PfsX15	ND	2.9	1.2	ND	ND	ND	IS*26*-*bla*_CTX-M-15/_*bla*_OXA-1_	Plasmid	MGE
	MB9635	68	p.E237X	p.S164VfsX12	ND	9.6	ND	ND	ND	ND	IS*26*-*bla*_CTX-M-27_;IS*26*-v1-*bla*_CTX-M-195_**	Plasmid	PCN
	MB6206	90	ND	ND	c.191_192insIS*Ecp1* TPU	8.6	ND	ND	ND	ND	IS*Ecp1*-*bla*_CTX-M-55_	Chromosome + plasmid	MGE + PCN
	MB5288	131	p.Y250X	p.Q88X	ND	5.4	4.7	ND	ND	ND	IS*26*-*bla*_CTX-M-15_/*bla*_OXA-1_	Chromosome	MGE
	MB2463	131	c.496_497insIS*26* TU	p.N183TfsX58	ND	0.8	17.7	ND	ND	ND	IS*26*-*bla*_CTX-M-15_/*bla*_OXA-1_	Chromosome	MGE
	MB8413	131	ND	p.N183TfsX58	ND	ND	14.9	ND	ND	ND	IS*26*-*bla*_OXA-1_	Chromosome	MGE
	MB2489	131	p.Y170KfsX2	p.F102TfsX6	ND	2.2	1.8	ND	ND	ND	IS*26*-*bla*_CTX-M-15_/*bla*_OXA-1_	Chromosome + plasmid	MGE + PCN
	MB9877	131	p.Q82X	ND	ND	9.1	ND	ND	ND	ND	IS*26*-*bla*_CTX-M-27_	Plasmid	MGE + PCN*
	MB9029	155	p.Q172X	p.A95AfsX75	ND	4.2	ND	2.6	ND	ND	IS*Ecp1*-*bla*_CTX-M-55_; IS*Ecp1*-*bla*_CMY-2_	Chromosome + plasmid	MGE + PCN
	MB6420	156	c.844_845insIS*10L*	c.44_45insIS*10L*	ND	ND	ND	ND	152.3	ND	Tn*2*-*bla*_TEM-1b_	Chromosome	MGE
	MB2791	167	c.908_909insIS*Ec35*	c.698_699insIS*Ec35*	ND	ND	ND	2.9	ND	ND	IS*IR*-*bla*_CMY-42_	Plasmid	PCN
	MB6066	405	ND	p.Y254EfsX3	ND	2.3	2.1	ND	ND	ND	IS*26*-*bla*_CTX-M-15_/*bla*_OXA-1_	Plasmid	PCN
	MB9880	405	ND	p.Y254EfsX3	ND	2.0	1.8	ND	4.0	ND	IS*26*-*bla*_CTX-M-15_/*bla*_OXA-1_;IS*26-v1*-*bla*_TEM-1b_	Plasmid	MGE + PCN
	MB2910	450	p.L32LfsX3	ND	ND	6.5	5.9	ND	ND	ND	IS*26*-*bla*_CTX-M-15_/*bla*_OXA-1_	Plasmid	MGE + PCN
CP*Ec*	MB3266	131	ND	ND	ND	ND	ND	ND	2.6	1.1	Tn*4401a-bla*_KPC-2_;IS*26*-*bla*_TEM-1b_	Plasmid	MGE
	MB9245	167	ND	ND	ND	2.1	1.9	ND	2.2	2.0	IS*26*-*bla*_NDM-5_;IS*26*-*bla*_CTX-M-15_/*bla*_OXA-1_;IS*26*-*bla*_TEM-1b_	Plasmid	PCN
	MB8134^*e*^	205	ND	c.1049_1050insIS2	ND	ND	ND	2.8	2.8	2.8	IS*3000*-*bla*_OXA-181_;*bla*_CMY-42_;*bla*_TEM-1b_	NA	NA
	MB8866^*e*^	361	ND	ND	ND	1.2	0.9	ND	ND	9.2	IS*1 × 1*-*bla*_OXA-232_;*bla*_CTX-M-15_	NA	NA
	MB5823	617	ND	ND	ND	1.2	ND	ND	ND	5.9	IS*26*-*bla*_NDM-5_;IS*Ecp1*-*bla*_CTX-M-15_	Chromosome + plasmid	MGE + PCN

a ND, not detected; MGE, mobile genetic element; PCN, plasmid copy number; NA, not applicable.

b Outer membrane porin mutations for frameshifts and insertions/deletions notated in amino acid mutation nomenclature. Otherwise, insertion sequence (IS) disruptions noted in nucleotide space. IS notated with ‘bp + 1 ATG’ indicate IS insertions upstream of outer membrane porin (*omp*) gene in promoter region.

c β-lactamase genes without preceding insertion sequence and NOT in the same mobilization unit (delimited by “/”) do not have sufficient genomic context for MGE estimate. **,Cannot differentiate copy number variants from homologs (i.e., genes with > 95% identity).

d Amp. pred. = amplification prediction. Plasmid copy number contributions were based on normalized coverage depths of full-length plasmid harboring β-lactamase = 1.5×, which indicates approximately 50% of the population has 2 copies of the β-lactamase-positive plasmid. *,MGE + PCN context resolved in part through extraction of individual long reads using SVants (74).

e Incomplete and/or short-read assemblies only preclude an estimate of genomic context of β-lactamase gene amplification.

**TABLE 2:** Summary of carbapenem nonsusceptibility mechanisms for K. pneumoniae with β-lactamase amplifications^*a*^

Carbapenem resistance status	Sample	MLST	*ompK36* ^*b*^	*ompK35* ^*b*^	ESBL CNV	OXA-1 CNV	TEM-1 CNV	CARB CNV	mGE + β-lactamase^*c*^	Genomic context	Amp. Pred.^*d*^
CI*Kp*	MB9017	307	p.D51RfsX37	ND	2.3	1.0	2.2	ND	IS*26*-*bla*_CTX-M-15_/*bla*_OXA-1_/*bla*_TEM-1b_	Chromosome	MGE
Non-CP-CR*Kp*	MB6487	15	IS*Ecp1* 18 bp +1 ATG	ND	11.4	ND	1.1	ND	IS*Ecp1*-*bla*_CTX-M-15_;Tn*2*-like transposon	Chromosome + plasmid	MGE
	MB3028	17	p.Q66X	ND	2.2	ND	ND	ND	IS*26*/IS*Ecp1*-*bla*_CTX-M-15_	Plasmid	PCN
	MB8190	29	p.Q310X	ND	5.5	6.4	4.2	ND	IS*Ecp1*-*bla*_CTX-M-15_/*bla*_OXA-1_/*bla*_TEM-1b_	Chromosome + plasmid	MGE
	MB9509	45	p.L305X	ND	8.5	ND	ND	ND	IS*903B*-*bla*_SHV-7_**	Plasmid	PCN
	MB2966	280	IS*Ecp1* TPU1 bp +1 ATG	ND	2.7	0.9	2.0	ND	IS*Ecp1*-*bla*_CTX-M-15_/*bla*_TEM-1b_;IS*26*-*bla*_OXA-1_	Chromosome + plasmid	MGE
	MB6013	307	p.D33EfsX4	p.W230X	5.7	4.9	1.0	ND	IS*26*-*bla*_CTX-M-15_/*bla*_OXA-1_;IS*26*-*bla*_OXA-1_	Plasmid	MGE
	MB4773	307	p.Q70X	ND	4.0	3.6	0.9	ND	IS*26*-*bla*_CTX-M-15_/*bla*_OXA-1_;IS*26-bla*_TEM-1b_	Plasmid	MGE
	MB3935	307	p.E87X	p.G208VfsX6	3.2	ND	1.1	ND	IS*Ecp1*-*bla*_CTX-M-15_;Tn*2*-like transposon	Plasmid	MGE
	MB6483	870	c.950_951insIS*26* TU	ND	5.0	ND	0.9	ND	IS*26*-*bla*_CTX-M-15_/*bla*_TEM-1b_IS*Ecp1*-*bla*_CTX-M-15_;	Chromosome + plasmid	MGE
	MB7964	2004	c.1115_1116insIS*Ecp1*TPU	ND	3.6	1.7	1.6	ND	IS*Ecp1*-*bla*_CTX-M-15_/*bla*_OXA-1_/*bla*_TEM-1b_	Chromosome + plasmid	MGE
	MB6413^*e*^	4060	p.A12AfsX11	ND	6.4	ND	ND	ND	IS*Ecp1*-*bla*_CTX-M-15_	NA	NA
CP*Kp*	KB346^*e*^	11	p.G134_D135dup	p.L28LfsX36	0.6 (CTX-M)6.1 (SHV)	0.7	0.5	0.6	Tn*4401a-bla*_KPC-2_;*bla*_CTX-M-65_;*bla*_SHV-12_;*bla*_OXA-1_;*bla*_TEM-1b_;	NA	NA
	MB9481	14	p.G134_D135dup	ND	3.3	0.5	ND	0.9 (NDM)0.8 (OXA-48)	Tn*125-bla*_NDM-1_;IS*Ecp1*-*bla*_CTX-M-15_;IS*Kpn26*-*bla*_OXA-48_;IS*26*-*bla*_OXA-1_	Chromosome + plasmid	MGE
	MB3242	15	ND	ND	ND	ND	ND	2.9	Tn*4401a-bla*_KPC-2_	Plasmid	PCN
	MB5951^*e*^	15	ND	ND	2.1	ND	1.8	2.0	Tn*4401a-bla*_KPC-2_;IS*Ecp1*-*bla*_CTX-M-15_;*bla*_TEM-1b_	NA	NA
	MB7231	29	ISKpn14 46 bp +1 ATG	ND	2.4	2.0	2.7	1.7	Tn*4401a-bla*_KPC-2;_IS*Ecp1*-*bla*_CTX-M-15_/*bla*_OXA-1_/*bla*_TEM-1b_	Chromosome + plasmid	MGE + PCN
	MB8806	307	p.G134_D135dup	p.G208VfsX6	2.3	ND	2.4	1.4	Tn*4401a-bla*_KPC-2;_IS*Ecp1*-*bla*_CTX-M-15_IS*26-bla*_TEM-1b_	Chromosome + plasmid	MGE

a ND, not detected; MGE, mobile genetic element; PCN, plasmid copy number; NA, not applicable.

*^b^*Outer membrane porin mutations for frameshifts and insertions/deletions notated in amino acid mutation nomenclature. Otherwise, insertion sequence (IS) disruptions are noted in nucleotide space. IS notated with “bp + 1 ATG” indicate IS insertions upstream of the outer membrane porin (*omp*) gene in the promoter region.

c β-lactamase genes without preceding insertion sequence and NOT in the same mobilization unit (delimited by “/”) do not have sufficient genomic context for MGE estimate. **,Cannot differentiate copy number variants from homologs (i.e., genes with > 95% identity).

d Amp. Pred. = amplification prediction. Plasmid copy number contributions were based on normalized coverage depths of full-length plasmid harboring β-lactamase = 1.5×, which indicates approximately 50% of the population has 2 copies of the β-lactamase positive plasmid.

e Incomplete and/or short-read assemblies only preclude an estimate of genomic context of β-lactamase gene amplification.

**FIG 4:**
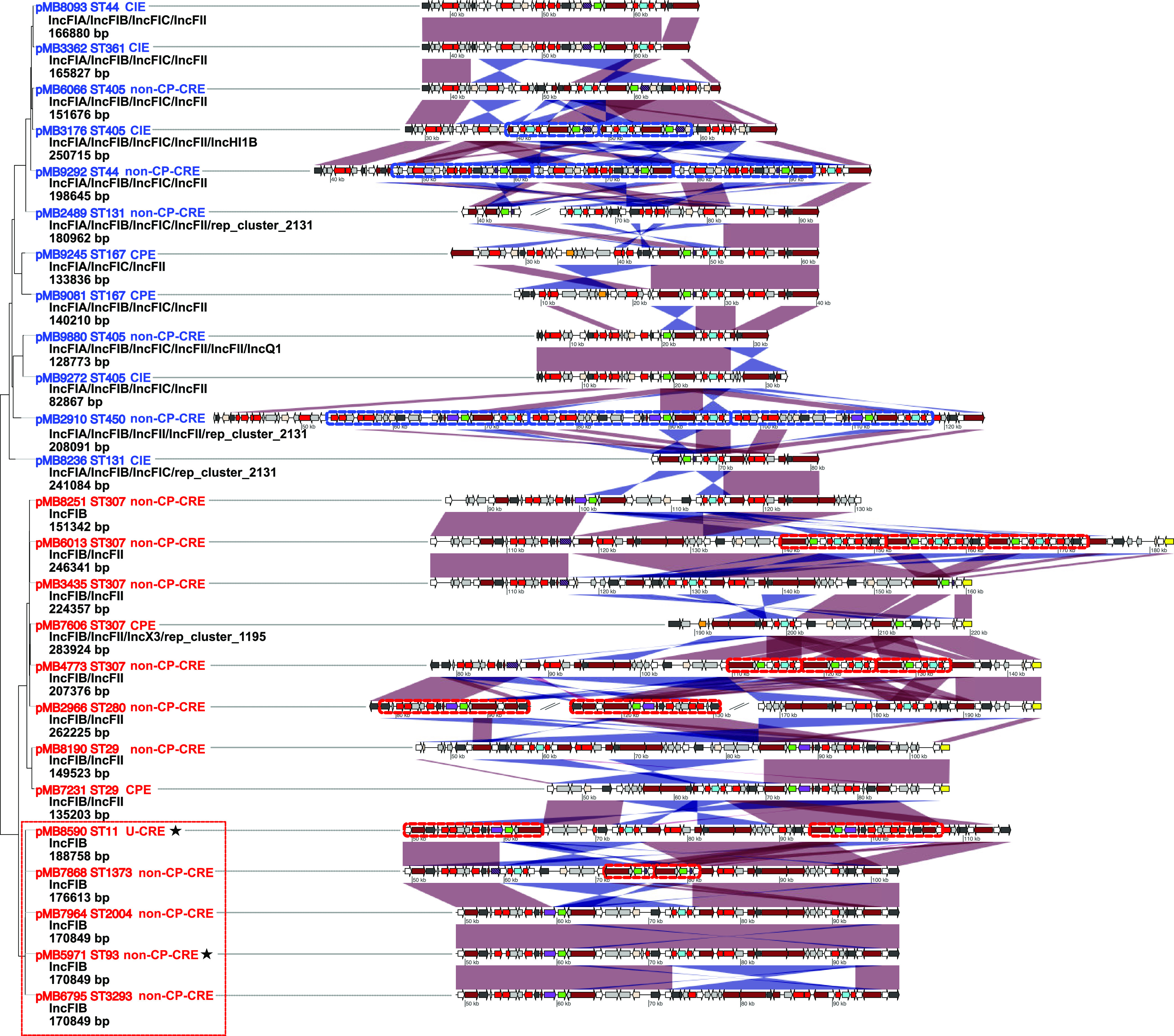
Multireplicon IncF-type plasmids cocarrying *bla*_CTX-M-15_ and *bla*_OXA-1_ shared across multiple *Enterobacterales* species. Neighbor-joining (NJ) tree based on estimated ANI pairwise distances of full-length, IncF-type multireplicon plasmids with red tip labels indicating Klebsiella spp. and blue tip labels indicating E. coli plasmids. Mobilization (MOB) typing designations with plasmid size are beneath each respective NJ tree tip label. Mobile genetic elements that have duplicated are demarcated on sequences with dotted lines colored by species (blue = E. coli; red = K. pneumoniae *spp*). Regions of plasmid are subset from each respective plasmid with position indicated on each structure to highlight the multidrug resistance region that includes *bla*_OXA-1_ (blue) and *bla*_CTX-M-15_ (green) open reading frame labels. Transposase/integrase (dark gray), IS*26* transposase (white), IS*26*-v1 (off-white), IS*Ecp1* transposase (purple), Tn3-like elements (brown), carbapenemases (orange), other antimicrobial genes (red), *rep* genes (yellow), and other genes (light gray) are labeled accordingly. Striped, purple IS*Ecp1* transposase ORFs indicate a disruption due to IS*26* or IS*26*-v1. The region on NJ tree enclosed by dotted red squares share ~99% identity and with three plasmids (pMB7964, pMB5971, and pMB6796) having ~99% coverage. Stars adjacent to tip labels indicate non-K. pneumoniae species (pMB5971 = K. aerogenes; pMB8590 = *K. michiganensis*). Linear comparisons between sequences indicate homology shared (min length = 1,000 bp, and >90% identity) in direct (red) and reverse (blue) orientation.

When considering the most commonly observed β-lactamase amplifications, we often detected the syntenic coupling on MGEs of *bla*_OXA-1_ and/or *bla*_CTX-M-15_ with frequent gene amplification either through a TPU or TU structure in CNS*Ec* (11/27; 41%) or CNS*Kp* (8/15;53%) as presented in Table 1 and 2, respectively. Indeed, when measuring binary presence/absence of β-lactamase genes in the entire CNSE cohort, 41% (32/79) of CNSE had *bla*_CTX-M-15_/*bla*_OXA-1_ cocarriage with both chromosomal and/or plasmid contexts, which is a comparable proportion to what has previously been reported in E. coli (38, 39). Of the 31 CNSE isolates that had ONT data available and *bla*_CTX-M-15_/*bla*_OXA-1_ cocarriage (one of the *bla*_CTX-M-15_/*bla*_OXA-1_-positive isolates only had a draft assembly), six isolates (three E. coli and three K. pneumoniae) had the two genes colocalized solely on the chromosome. The majority of *bla*_CTX-M-15_/*bla*_OXA-1_ colocalization was observed in a plasmid context (81%; 25/31) with all but one CNSE isolate (MB5646) having cocarriage on multireplicon IncF-type plasmids. Therefore, we calculated an estimate of pairwise average nucleotide identity (ANI) of all IncF-type plasmids harboring *bla*_CTX-M-15_*/bla*_OXA-1_ (Fig. 4) to determine the relatedness of these IncF-type plasmids and see if there was evidence of interclade and interspecies transmission. A full-length visualization of the multireplicon IncF-type plasmids can be found on Fig. S3.

10.1128/msystems.00476-22.9 FIG S3 Full-length linear comparisons of IncF-type plasmids. Transposases/integrases (dark grey), IS*26* transposase (white), IS*26*-v1 (off-white), IS*Ecp1* transposase (purple), carbapenemases (orange), other antimicrobial genes (red), *rep* genes (yellow), *bla*_OXA-1_ (blue arrow), *bla*_CTX-M-15_ (green arrow), virulence factors (pink), and other genes (light grey) are labelled accordingly. Striped IS*Ecp1* transposase ORFs indicate a disruption due to (IS*26* or IS*26*-v1). Linear comparisons between sequences indicate homology shared (min length = 1,000 bp, and 90% identity) in direct (red) and reverse (blue) orientation. Download FIG S3, PDF file, 0.6 MB. Copyright © 2022 Shropshire et al. 2022 Shropshire et al. https://creativecommons.org/licenses/by/4.0/ This content is distributed under the terms of the Creative Commons Attribution 4.0 International license.

The ANI of all *bla*_CTX-M-15_*/bla*_OXA-1_-positive IncF-type plasmids was highly similar (average = 0.94; SD = 0.04) across E. coli (*n* = 12) and Klebsiella spp. (*n* = 13) with two primary clusters that formed by species when observing the neighbor joining distance inferred dendrogram (Fig. 4). The discrimination between E. coli and K. pneumoniae IncFIB plasmids was largely due to differences in transmission of well-characterized replication initiation protein alleles found in Klebsiella spp. (i.e., IncFIB_K_) and E. coli (i.e., IncFIB [AP001918]). One nested cluster of five IncFIB plasmids demarcated by a red box in Fig. 4 shared >99.9% ANI across three unique K. pneumoniae STs (pMB7868_1, pMB7964_1, and pMB6795_1), K. aerogenes (pMB5971_1), and *K. michiganensis* (pMB8590_1). Interestingly, we observed *bla*_CTX-M-15_ and/or *bla*_OXA-1_ amplification occurring on 8/25 (32%) plasmids (Fig. 4; black striped boxes) with all but one plasmid (pMB2966_1) having an IS*26*-mediated TU amplification. Out of the seven TUs with TU amplification, six were tandem arrays, whereas only one plasmid (pMB8590_1) had a segmental duplication (i.e., mobilization to another genomic context) (Fig. 4).

**FIG 5:**
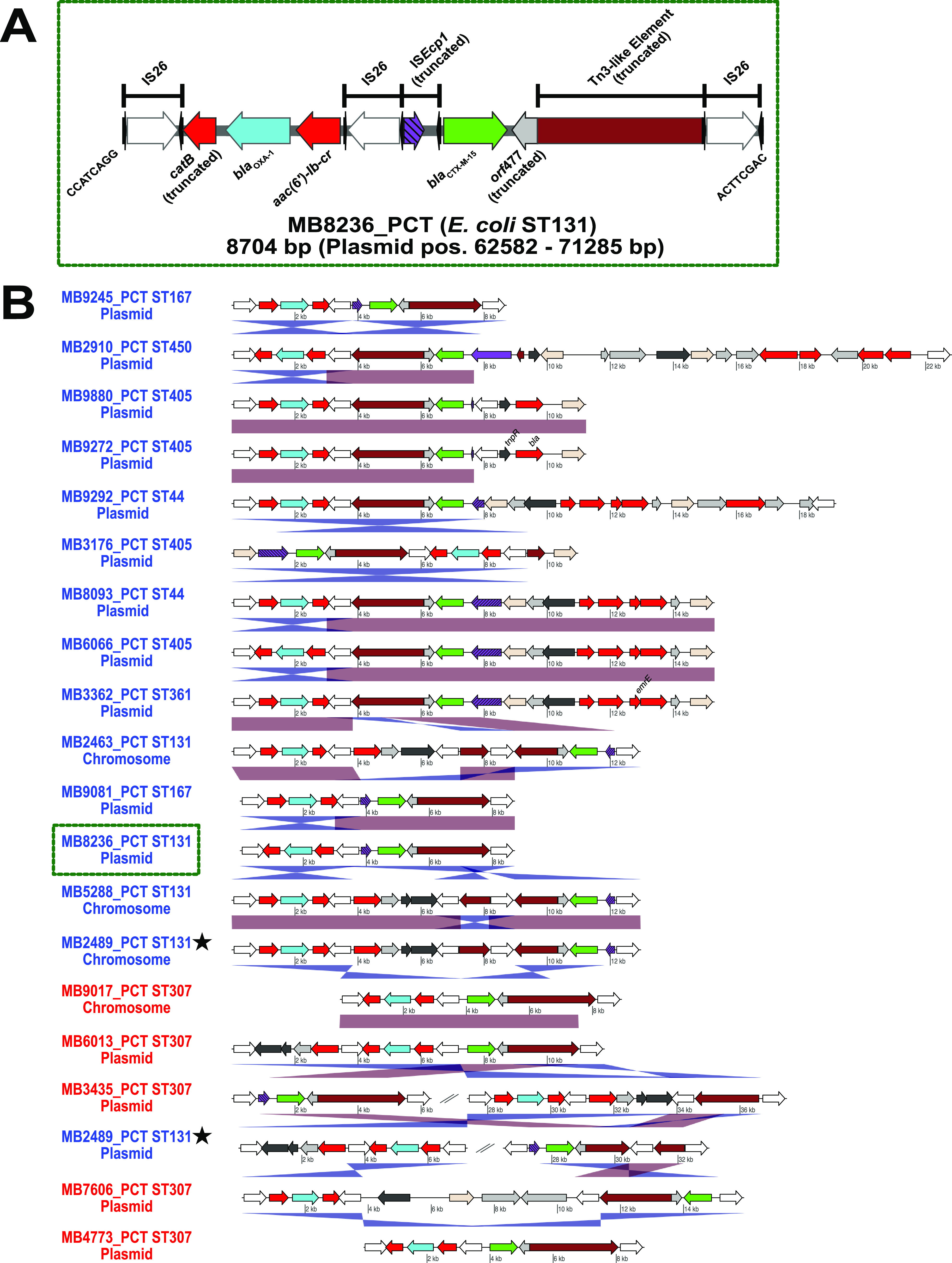
Pseudocompound transposons (PCTs) driving mobilization and amplification of ESBL and narrow-spectrum β-lactamases. Transposase/integrase (dark gray), IS*26* transposase (white), IS*26*-v1 (off-white), IS*Ecp1* transposase (purple), Tn3-like elements (brown), other antimicrobial genes (red), *bla*_OXA-1_ (blue), *bla*_CTX-M-15_ (green), and other genes (light gray) are labeled accordingly. Striped, purple IS*Ecp1* transposase ORFs indicate a disruption due to IS*26* or IS*26*-v1. (A) Representation of pseudocompound transposon (MB8236_PCT) flanked by IS*26* in direct orientation within a plasmid context. Black arrows flanking IS transposases indicate inverted repeats. There is an 8-bp DNA flanking IS*26* on linearized representation of PCT. Position on plasmid is indicated in parenthesis. (B) Plasmid and chromosomal contexts of PCT within E. coli (blue) and K. pneumoniae (red) indicating blastn identities as described in Fig. 4. Stars indicate PCTs arising from the same genome. Green dotted line highlights the PCT that is fully annotated in (A). Linear comparisons between sequences indicate homology shared (min length = 1,000 bp, and >90% identity) in direct (red) and reverse (blue) orientation.

We next sought to characterize and distinguish the IS*26*- and IS*Ecp1*-mediated mechanisms that were responsible for mobilizing *bla*_CTX-M-15_*/bla*_OXA-1_ from both a plasmid and chromosomal context. Fig. 5A provides an illustration of a pseudocompound transposon (PCT) that can be made of two or more IS*26* units, which must include flanking IS*26* transposase in direct orientation for potential cointegrate formation to occur and mobilize the passenger AMR genes (40). Fig. 5B shows the highly modular mosaic structures of these PCTs, except for one PCT (MB2910_PCT), include an IS*26* or IS*26*-v1 element upstream of *bla*_CTX-M-15_, disrupting the IS*Ecp1* ORF. Interestingly, these PCTs with disrupted IS*Ecp1* were more commonly observed in E. coli than in K. pneumoniae, apart from five ST307 K. pneumoniae isolates (Fig. 5B). There was only one isolate (MB2489) with a likely chromosome-to-plasmid IS*26* transposase-mediated cointegration formation event (Fig. 5B) based on chromosomal gene content present on the plasmid (41, 42).

**FIG 6:**
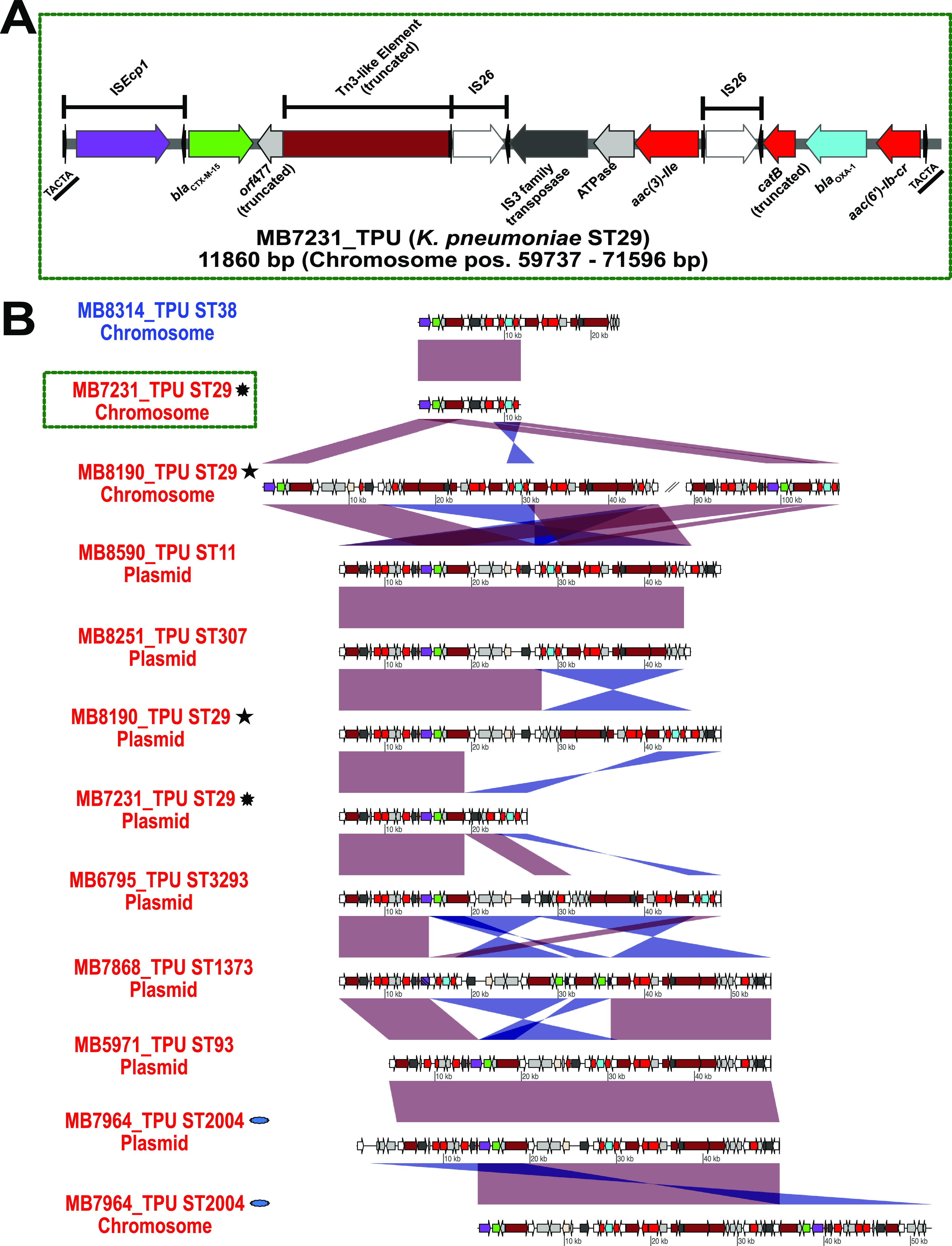
Transposition units (TPUs) driving mobilization and amplification of ESBL and narrow-spectrum β-lactamases. Transposase/integrase (dark gray), IS*26* transposase (white), IS*26*-v1 (off-white), IS*Ecp1* transposase (purple), Tn*3*-like elements (brown), other antimicrobial genes (red), *bla*_OXA-1_ (blue), *bla*_CTX-M-15_ (green), and other genes (light gray) are labeled accordingly. Striped, purple IS*Ecp1* transposase ORFs indicate a disruption due to IS*26*. (A) Example of K. pneumoniae chromosomal context of transposition unit (MB7231_TPU) mobilized from plasmid to chromosome via IS*Ecp1.* Black arrows flanking IS transposases indicate inverted repeats. A 5-bp direct repeat (underlined) flanking MB7231_TPU is indicated on end of the linearized representation of TPU. Position on chromosome indicated in parenthesis. (B) Plasmid and chromosomal contexts of TPU within E. coli (blue) and K. pneumoniae (red) indicating blastn identities as described in Fig. 4. Matching symbols adjacent to labels indicate TPUs arising from the same genome. The green dotted line highlights the TPU that is fully annotated in (A). Linear comparisons between sequences indicate homology shared (min length = 1,000 bp, and >90% identity) in direct (red) and reverse (blue) orientation.

The other common MGE with the potential to mobilize *bla*_CTX-M-15_*/bla*_OXA-1_ was IS*Ecp1*-mediated transposable units (TPUs). Indeed, Fig. 6A provides a schematic for a representative K. pneumoniae TPU (MB7231_TPU) found in a chromosomal context. In contrast to CNS*Ec*, 53% of FIB Klebsiella spp. plasmids had intact IS*Ecp1* immediately upstream of *bla*_CTX-M-15_ suggesting the potential for TPU formations as the primary driver of *bla*_CTX-M-15_ mobilization in non-ST307 CNS*Kp* (Fig. 6B). There were three CNS*Kp* isolates that had plasmid-to-chromosome transfer of IS*Ecp1*-mediated TPUs, as detected by 5 bp target site duplications flanking the inverted repeat regions of the chromosomal TPUs (Fig. 6B). Taken together, our analysis highlights the enrichment of IS*26*/IS*Ecp1* structures present in CNSE that have a strong association with amplifications of β-lactamase genes, in particular, *bla*_CTX-M-15_ and *bla*_OXA-1_ in our cohort.

### Characterization of unconfirmed nonsusceptible *Enterobacterales* (U-CNSE) isolates.

In light of the increasing recognition of the impact of unconfirmed CRE (1), we next sought to characterize a subset of E. coli and Klebsiella spp. for which we had not confirmed carbapenem nonsusceptibility to our non-CP-CNSE isolates. In marked contrast to non-CP-CR*Ec* and non-CP-CR*Kp* isolates, none of the U-CNS E. coli (*n* = 3) and Klebsiella spp. (*n* = 2) had mutated OmpC/OmpF (OmpK36/OmpK35)-encoding genes (Fig. 2; Table S2). All the U-CNS E. coli and Klebsiella spp. were *bla*_CTX-M_-positive (4 *bla*_CTX-M-15_; 1 *bla*_CTX-M-55_); furthermore, amplification of ESBL encoding enzymes were detected (median ESBL CNV = 2.4×) among all five. U-CNS E. coli and Klebsiella spp. β-lactamase gene amplification in the U-CNS isolates shared similar mechanisms to that observed for the non-CP-CRE strains. For example, MB8590 (*K. michiganensis*; ST11) had evidence of a plasmid TU harboring *bla*_CTX-M-15_*/bla*_OXA-1_ that had two copies via segmental duplication (Fig. 4). Furthermore, this TU included an intact IS*Ecp1* (Fig. 6B) suggesting the potential for TPU-mediated mobilization as well. Although a small number of isolates were examined, these data indicate that intact porins are the major distinction between unconfirmed and CNS E. coli and Klebsiella spp.

## DISCUSSION

Through a comprehensive, comparative genomics analysis on a diverse array of CNSE bacteremia isolates, we expanded the current understanding of the breadth of MGE-mediated mechanisms used to overcome carbapenems in clinically important *Enterobacterales* strains. By analyzing normalized coverage depths of β-lactamase-encoding genes in conjunction with the detection of binary presence/absence of β-lactamases and *omp* genes, we show that amplification of ESBL genes as well as disruption of *omp* genes are commonly found among invasive non-CP-CRE. Additionally, our ONT long-read sequencing data allowed for full characterization of the complex MGE-mediated gene amplifications and genetic alterations that can generate carbapenem resistance in the absence of a carbapenemase. The increasing appreciation of both the scope and clinical impact of non-CP-CRE (1, 2) highlights the need to develop novel diagnostic and therapeutic strategies for this understudied group of organisms.

A key finding was the high prevalence of CNSE organisms that lacked carbapenemases with non-CP strains accounting for well over 70% of both CNS E. coli and K. pneumoniae in our cohort. One possible explanation for this finding was our inclusion of organisms with carbapenem-intermediate susceptibility phenotypes (i.e., CIE strains), a decision which was based on the recent CRACKLE-2 finding that patients with unconfirmed CNSE, which often tested intermediate to ertapenem or other carbapenems, had similar clinical outcomes to patients with confirmed CRE (1). Given that carbapenem MICs tend to be lower for non-CP-CRE versus CPE (1, 34, 43), our inclusion of CIE strains likely increased our proportion of non-CP isolates. However, even when only CRE isolates were considered, we still observed a predominance of non-CP organisms for both E. coli (19/25; 76%) and K. pneumoniae (18/26; 69%). Whereas a high percentage of non-CP-CR*Ec* strains has consistently been found in CRE surveillance studies, the opposite is true of K. pneumoniae where the high prevalence of *bla*_KPC_ typically results in >70 to 80% of CR*Kp* organisms being carbapenemase-positive in the United States (1, 34). The high percentage of non-CP-CRE in our cohort was particularly interesting given that we only examined bacteremia isolates which are sufficiently fit to cause a serious infection inasmuch as non-CP-CRE isolates are often considered to have a fitness defect relative to CPE strains (44–46). The reasons underlying the high prevalence of non-CP isolates in our bacteremia cohort are not currently known but may include relatively stringent infection control practices among our highly immunocompromised patients. Recently, Black et al. noted a higher prevalence of non-CP-CRE (59%) in south Texas where non-CP-CRE patients were more likely to receive a longer duration of antibiotic treatment as well as more likely to have an emergency department visit compared to CPE, albeit with low number of observations (47). This finding is consistent with our cancer patient population that receives a high level of antibiotic treatment (48) and coincides with the finding that previous antibiotic exposure has been identified as a risk factor for non-CP-CRE relative to CPE in other studies (7).

The high percentage of non-CP organisms in our cohort led us to focus on using our genomic data to better understand mechanisms driving carbapenem resistance in the absence of a carbapenemase. There were several important findings from these analyses. First, consistent with previous data based primarily on laboratory studies of passaged strains and PCR-based methods (24–26), we found that non-CP-CRE almost always had combined porin disruption and amplification of ESBL-encoding genes. While many studies have documented how an increase in AMR gene copy number corresponds to an increased AMR phenotype (13–17, 33), to our knowledge, our study is the first to systematically demonstrate an ESBL gene copy number increase in a large cohort of non-CP-CRE bacteremia isolates. It is thought that the porin disruption limits carbapenem penetration into the periplasm to the point where high level ESBL production can inactivate sufficient carbapenem to generate resistance (4). Thus, incorporating porin assessment and β-lactamase gene amplification could assist with predicting *Enterobacterales* carbapenem susceptibility using genomic data (49–51). Second, the non-CP-CRE isolates were genetically heterogenous and primarily encoded various CTX-M-type ESBLs with or without OXA-1. ESBL variants of TEM or SHV were quite rare in E. coli (*n* = 2) and K. pneumoniae (*n* = 3), as was plasmid-borne AmpC in E. coli (*n* = 5) and K. pneumoniae (not detected). These findings may reflect the dominant nature of CTX-M-containing strains among ESBL isolates and are congruent with a previous laboratory study indicating multiple classes of CTX-M enzymes can reduce ertapenem susceptibility under selective pressure in porin deficient backgrounds (26). Finally, we observed minimal clonality among the non-CP-CRE strains indicating that the organisms developed carbapenem resistance independently rather than being transmitted between patients. This hypothesis is supported by our observation that in many of the non-CP-CR E. coli and K. pneumoniae cases, the patients had previously had a bloodstream infection with an ESBL-producing carbapenem-susceptible organism. Thus, it is highly likely that carbapenem treatment of the ESBL infection selected for non-CP-CRE strains via ESBL amplification and porin disruption. Given that in our previous study only a small percentage of patients treated for an ESBL infection subsequently developed a non-CP-CRE infection (13), we are actively investigating why particular genetic backgrounds may contribute to a higher probability of developing carbapenem resistance versus other ESBL-positive *Enterobacterales* strains.

The use of ONT sequencing was critical in helping to delineate the diverse MGE mechanisms underlying increases in ESBL gene copy numbers, which in general are not discernible with the commonly used short-read, whole-genome sequencing or PCR-based approaches (19). The vast majority of the ESBL amplifications involved CTX-M encoding genes with long-read data, indicating that these amplifications were likely due to IS*26* translocatable units or IS*Ecp1* transposition units increasing in copy via segmental duplication or *in situ* tandem amplification. Both IS*26* and IS*Ecp1* contain transposases capable of mobilizing AMR genes (albeit very different mechanisms), with IS*26*-mediated gene amplification increasingly recognized as a cause of progressive resistance to various β-lactams (13, 15, 20, 41, 42, 52). The complex MGEs amplified by IS*26* and IS*Ecp1* often contained non-β-lactamase-encoding genes that confer resistance to aminoglycosides (e.g., *aac[6’]-Ib-cr*), tetracyclines (e.g., *tetAR*), trimethroprim (e.g., *dfrA17*), and sulfonamides (e.g., *sul1*) as illustrated in Fig. 4 and 6. Therefore, similar to CPE, our non-CP-CRE was often multidrug resistant (Table S4), further hindering treatment options. Another finding of concern was identifying IS*26* or IS*Ecp1* coamplification of two β-lactamases on the same transposable unit (Table 1 and 2), typically *bla*_CTX-M-15_ along with *bla*_OXA-1,_ but also *bla*_CTX-M-15_ with *bla*_CMY-4_ and *bla*_CTX-M-55_ with *bla*_CMY-2_. These dual β-lactamase-encoding gene amplified organisms often were nonsusceptible to meropenem in addition to ertapenem (Table S4).

Our findings along with other data (30, 31, 51) suggest carbapenem-nonsusceptible *Enterobacterales* reside along a spectrum mediated to a major degree by changes in porin function and β-lactamase gene copy number. It is likely that unconfirmed CNSE consist of a heterogenous population of ESBL-positive, carbapenem-adapting strains with β-lactamase gene amplifications/porin disruptions which may give different phenotypic results depending on the colony tested (32). Further carbapenem adaptation may fix a single porin disruption as seen in our E. coli ST405 isolates in Fig. 2, and/or increase β-lactamase gene copy number within the population, leading to a carbapenem-intermediate phenotype that progresses to full resistance through further β-lactamase amplification and concurrent outer membrane porin disruption. This progressive β-lactam resistance model is analogous to that recently identified for *bla*_TEM-1_ and *bla*_OXA-1_ amplifications mediating piperacillin-tazobactam resistance (13, 15, 16, 33). The increasing rates of ESBL-positive *Enterobacterales* infections means that there are growing opportunities for development of non-CP-CRE. Given the widespread nature of IS*26*-mediated TUs and IS*Ecp1-*mediated TPUs in association with ESBL enzymes, our data suggest that optimizing carbapenem therapy (choice of carbapenem, dose, and duration) of ESBL infections is likely to be critical to minimizing non-CP-CRE emergence.

Our study has some inherent limitations. First, we only assayed strains from a gDNA context. It is likely that non-CP-CRE mechanisms also include transcriptional and posttranscriptional changes that we did not discern. However, there were only a few CNSE strains where a DNA-based explanation for an observed phenotype could not be identified, and these strains will be assessed using other methodologies as part of future studies. Second, we focused on particular genomic areas, specifically, known β-lactamase-encoding elements and porin-encoding genes. Thus, it remains possible that other, yet to be identified, DNA alterations contributed to the carbapenem susceptibility phenotypes. Similarly, we did not recreate the DNA modifications of interest in an isogenic background to conclusively demonstrate that the identified changes conferred carbapenem resistance. However, our findings are in line with those derived from previous laboratory passaged and genetically altered strains (24–26). Finally, given the large number of sequenced isolates, we did not assess for population heterogeneity, the impact of which we attempted to minimize by performing phenotypic and genotypic analyses on the same single colony.

In summary, we present a cohort of fully resolved genomes of carbapenem-nonsusceptible *Enterobacterales* causing invasive infections, focusing on a large number of noncarbapenemase-producing E. coli and K. pneumoniae isolates. Our data shed light on the pleiotropic and potentially widespread mechanisms underlying the non-CP-CRE phenotype and suggest that antimicrobial stewardship practices are likely to be critical in efforts to decrease non-CP-CRE impact.

## MATERIALS AND METHODS

### Study design.

Our lab has a comprehensive storage of The University of Texas MD Anderson Cancer Center (MDACC) bacteremia isolates (i.e., the Microbe Bank Database [MBD]) dating back to 2012 stocked at −80°C in thioglycolate media with 25% glycerol. CLSI 2018 M100 guidelines were used to determine MIC breakpoint interpretations for carbapenem resistance (53). *Enterobacterales* bacteremia isolates (*n* = 143) with a nonsusceptible MIC interpretation to ertapenem (ETP) (>0.5 μg/mL) or meropenem (MEM) (>1 μg/mL) as reported by the MDACC Division of Pathology and Laboratory Medicine (PLM) clinical microbiology laboratory were selected using the Epic EHR software workbench reporting tool from July 1st, 2016, to June 30th, 2020. *Enterobacterales* species with intrinsic resistance to carbapenems (e.g., Proteus mirabilis) were excluded from selection. Candidate isolates underwent additional MIC testing to confirm ETP nonsusceptibility as identified by the PLM lab using Etest (bioMérieux) gradient MIC strips. Definitions of carbapenem nonsusceptibility were based on the following criterion: (1) carbapenemase-producing *Enterobacterales* (CPE) = carbapenemase detection confirmed through whole-genome sequencing (WGS); (2) noncarbapenemase-producing carbapenem-resistant *Enterobacterales* (non-CP-CRE) = no carbapenemase detected in WGS with confirmation Etest ETP MIC ≥ 2 μg/mL *and* MDACC ETP MIC ≥ 2 μg/mL, *or* MEM MIC ≥ 4 μg/mL; (3) carbapenem intermediate *Enterobacterales* (CIE) = (a) confirmation Etest 0.5 μg/mL < ETP MIC < 2.0 μg/mL, or (b) MDACC MIC where 0.5 μg/mL < ETP MIC < 2.0 μg/mL *or* 1 μg/mL < MEM MIC < 4.0 μg/mL; (4) unconfirmed carbapenem nonsusceptible *Enterobacterales* (U-CNSE) = confirmation Etest ETP MIC ≤ 0.5 μg/mL.

CNSE exclusion criteria included isolates not available in the MBD (*n* = 10), serial isolates (i.e., any consecutive, recurrent bacteremia isolate with identical species as identified by the PLM lab) (*n* = 25), isolates from same culture (*n* = 4), and U-CNSE phenotype isolates and/or isolates with no growth on ertapenem (0.5 μg/mL) supplemented THY agar (*n* = 25). The first available ETP-nonsusceptible isolate per patient from the MBD that met the above definition and the screening process, was selected for whole-genome sequencing. There were two isolates, MB8134 and MB8251, with differential *Enterobacterales* species cultured from the same patient and isolated 18 days apart, that were included in the total CNSE cohort. After screening for carbapenem nonsusceptibility from available isolates (see Fig. 1), our sampling frame resulted in 79 total CNSE isolates that were sequenced from 78 unique patients. In addition to our CNSE WGS cohort, we performed WGS on 8 U-CNSE to investigate unstable carbapenem-nonsusceptible phenotypes. An antibiogram of the 79 CNSE isolates + 8 U-CNSE isolates is available on Table S4.

### Illumina short-read and Oxford Nanopore Technologies long-read sequencing.

All isolates were streaked from the MBD collection and grown on THY overnight at 37°C. Single colonies were picked and grown in LB broth for 4 h at 37°C with mild agitation and subsequently a pellet was stored at −80°C until gDNA extraction. The extraction of gDNA was performed using the MasterPure Complete DNA and RNA purification kit using manufacturer’s instructions. Genomic DNA concentration was measured using the Qubit 4 fluorometer with complementary measurement of concentration and A260/280; A260/230 performed on an Eppendorf BioPhotometer. Isolates were then library prepped using the Illumina DNA Prep kit and sequenced using the Illumina NovaSeq 6000 platform. Select isolates were then sequenced using the long-read Oxford Nanopore Technologies (ONT) GridION platform with the Rapid Sequencing kit (SQK-RAD004) per manufacturer’s instructions.

Short-read Illumina fastq data were trimmed, quality checked, and assembled using a customized workflow (Shropshire W, SPAdes_pipeline-v0.1.0-alpha, GitHub: https://github.com/wshropshire/SPAdes_pipeline) with assemblies generated using SPAdes v3.15.3 using the “—isolate” parameter in addition to default parameters for paired-end short-read data. Short-read and long-read data were used with the Flye v2.9-b1768 assembler pipeline (Shropshire, W.; flye_hybrid_assembly_pipeline-v0.3.0-alpha; https://github.com/wshropshire/flye_hybrid_assembly_pipeline). Genome assembly quality was assessed with CheckM v1.2.0 (54) with mean coverage depth of complete and draft assemblies calculated using mosdepth v0.3.3 (55). An overview of genome assembly quality metrics is presented on Table S6.

10.1128/msystems.00476-22.6 TABLE S6 Long-read sequencing data by individual isolate. Download Table S6, XLSX file, 0.02 MB. Copyright © 2022 Shropshire et al. 2022 Shropshire et al. https://creativecommons.org/licenses/by/4.0/ This content is distributed under the terms of the Creative Commons Attribution 4.0 International license.

### Pan genome and maximum likelihood (ML) phylogenetic analysis.

Complete and draft assemblies were then used as input for pan genome analysis using Panaroo v.1.2.9 (56) using the moderate --clean-mode parameter with the mafft core gene alignment option. This core gene alignment file was then used as input to create a maximum-likelihood phylogenetic tree with IQTree2 v2.2.0-beta (57). When creating the core gene inferred ML phylogenetic tree, model selection was performed using ModelFinder (58), a nonparametric bootstrap approximation, UFBoot (59) (*n* = 1,000), and an SH-aLRT (*n* = 1,000) test to further evaluate branch lengths. Tree visualization along with the addition of metadata was completed using ggtree v3.1.1 and ggtreeExtra v1.0.4, respectively. Clustering of isolates based on core gene alignment was assessed using the rhierhaps-1.1.3 tool (60). Pairwise SNP differences were assessed using the snp-dists tool (Seemann, T.; snp-dists-v0.8.2; https://github.com/tseemann/snp-dists).

### Antimicrobial resistance genes and *in silico* typing profiles.

Kleborate v2.0.4 (61) was used with draft and complete assemblies to identify K and O antigen profiles (Kleborate confidence scores of “Good” or better), MLST, acquired and chromosomal antimicrobial resistance, and virulence factors for isolates belonging to the Klebsiella pneumoniae species complex (KpSC). Additionally, Kleborate (61) was used to designate species taxa for all isolates sequenced by calculating pairwise Mash distances (62) between each respective genome assembly and their Enterobacterales reference genomes (*n* = 2,619). All isolates had strong species matches (i.e., Mash distances < 0.02). SerotypeFinder v2.0 (63) was used for *in silico* serotyping of E. coli isolates using an 85% blastn identity/60% minimum length threshold for O and H antigen identification using complete or draft assemblies. Novel MLST schema not identified using Kleborate v2.0.4 or the mlst v2.19.0 Perl script (Seemann, T.; mlst-2.19.0; https://github.com/tseemann/mlst) was identified using the MLST v2.0 server (64). Phylogroups of E. coli were detected using the ClermonTyping v20.03 tool (65) using the clermonTyping.sh script. The BLASTn alignment tool (BLAST 2.11.0+) was used with an in-house database of E. coli *ompC* and *ompF* genes (MG1655 K-12 reference) and their respective enterobacterial homologs identified in Klebsiella spp., Enterobacter spp., *Citrobacter* spp., and Serratia marcescens to characterize potential osmoporin gene disruption. SnapGene v5.0.8 was used to visualize these osmoporin gene disruptions and further characterize MGE-associated insertions within the open reading frame and/or promoter region using ISFinder (66).

### AMR gene and plasmid copy number variation estimation.

Antimicrobial resistance genes were detected using the KmerResistance v2.2.0 (67, 68) tool which uses KMA-1.3.24a to detect AMR genes using a short-read k-mer-based alignment against the ResFinder (Accessed 5 November 2021). These ResFinder hits were then used as input for a copy number variant estimation tool (Shropshire, W.; convict-v1.0; https://github.com/wshropshire/convict), which estimates gene copy number variants by normalizing coverage depths to housekeeping genes. Core genes present in >99% of the consensus, pan genome fasta file generated from Panaroo were used to control coverage depth (i.e., 3211 core genes). We only reported AMR gene copy number variants with 100% coverage and 100% identity as reported through KmerResistance. We performed qPCR for further validation of CONVICT with one high and low CNV *bla*_CTX-M-15_/*bla*_OXA-1_ sample (MB5288 and MB8093, respectively) with results presented on Fig. S4.

10.1128/msystems.00476-22.10 FIG S4 Comparison of qPCR with CONVICT read mapping depth data. qPCR was performed using genomic DNA from Escherichia coli strains MB5288 (strain with amplified *bla*_CTX-M-15_ and *bla*_OXA-1_ as measured via CONVICT) and MB8093 (strain containing nonamplified *bla*_CTX-M-15_ and *bla*_OXA-1_ as measured via CONVICT). Strains were assayed using TaqMan qPCR as described in (Shropshire WC, Aitken SL, Pifer R, Kim J, et al. J Antimicrob Chemother 76:385–395, 2021, https://doi.org/10.1093/jac/dkaa447) in triplicate with C_T_ values normalized to the endogenous control gene *rpsL* and data presented being mean ± standard deviation. For both qPCR (red bars) and CONVICT (blue bars), data presented are log_2_ ratio of MB5288 relative to MB8093 for indicated gene. Download FIG S4, EPS file, 0.9 MB. Copyright © 2022 Shropshire et al. 2022 Shropshire et al. https://creativecommons.org/licenses/by/4.0/ This content is distributed under the terms of the Creative Commons Attribution 4.0 International license.

SVants (Hanson, B.; GitHub: https://github.com/EpiBlake/SVants) was used to confirm copy number variants with individual ONT long-reads containing multiple tandem repeats of IS*26* and IS*Ecp1* multiresistance determinant regions for isolates with increased coverage depth mapping visualized in IGV-2.9.4. A ratio of mean coverage depths of plasmid-to-chromosome was calculated using bwa mem alignments and the pileup.sh script from bbmap-v38.79 to get an approximation of plasmid copy number (PCN).

Plasmid typing of completed assemblies was completed using the mob_typer-v3.0.0 command line tool (69). FastANI-v1.31 (70) was used to estimate average nucleotide identity across plasmid and MGE structures with default settings. The bacsort script (Wick, R.; GitHub: https://github.com/rrwick/Bacsort), “pairwise_identities_to_distance_matrix.py” is used to convert FastANI pairwise distances to a distance matrix in PHYLIP format with a maximum genetic distance of 0.20. This distance matrix was used as input to create a neighbor-joining tree using the BIONJ algorithm (71) using the ape-v.5.6-1 R package (72). Genome comparisons and annotations of plasmid and MGE structures was performed using the genoPlotR-v0.8.11 R package (73). In order to filter multiple IS comparisons, a minimum sequence fragment length of 1000 bp was used to compare blastn identities ≥90% in direct (red) or reverse (blue) orientation.

### Statistics.

All statistics were performed using R v4.0.4 (15 February 2021). Significant increases in AMR gene copy numbers were assessed using one-sample Wilcoxon tests with a one-sided alternative hypothesis that mean CNV was greater than 1. Scatterplot and boxplots were generated using ggplot2 v3.3.5.

### Data availability.

Short-read Illumina data, long-read ONT data, as well as complete and draft assemblies are available in the NCBI BioProject repository (PRJNA836696). Three samples (MB2315, MB2446, MB2463) have data available from a previous BioProject (PRJNA603908).
