# Supplementary figures and images for: Systematic Analysis of Mobile Genetic Elements Mediating β-Lactamase Gene Amplification in Noncarbapenemase-Producing Carbapenem-Resistant Enterobacterales Bloodstream Infections
Source: mSystems. 2022 Aug 29;7(5):e00476-22. doi: 10.1128/msystems.00476-22 (PMC9601100; doi:10.1128/msystems.00476-22)

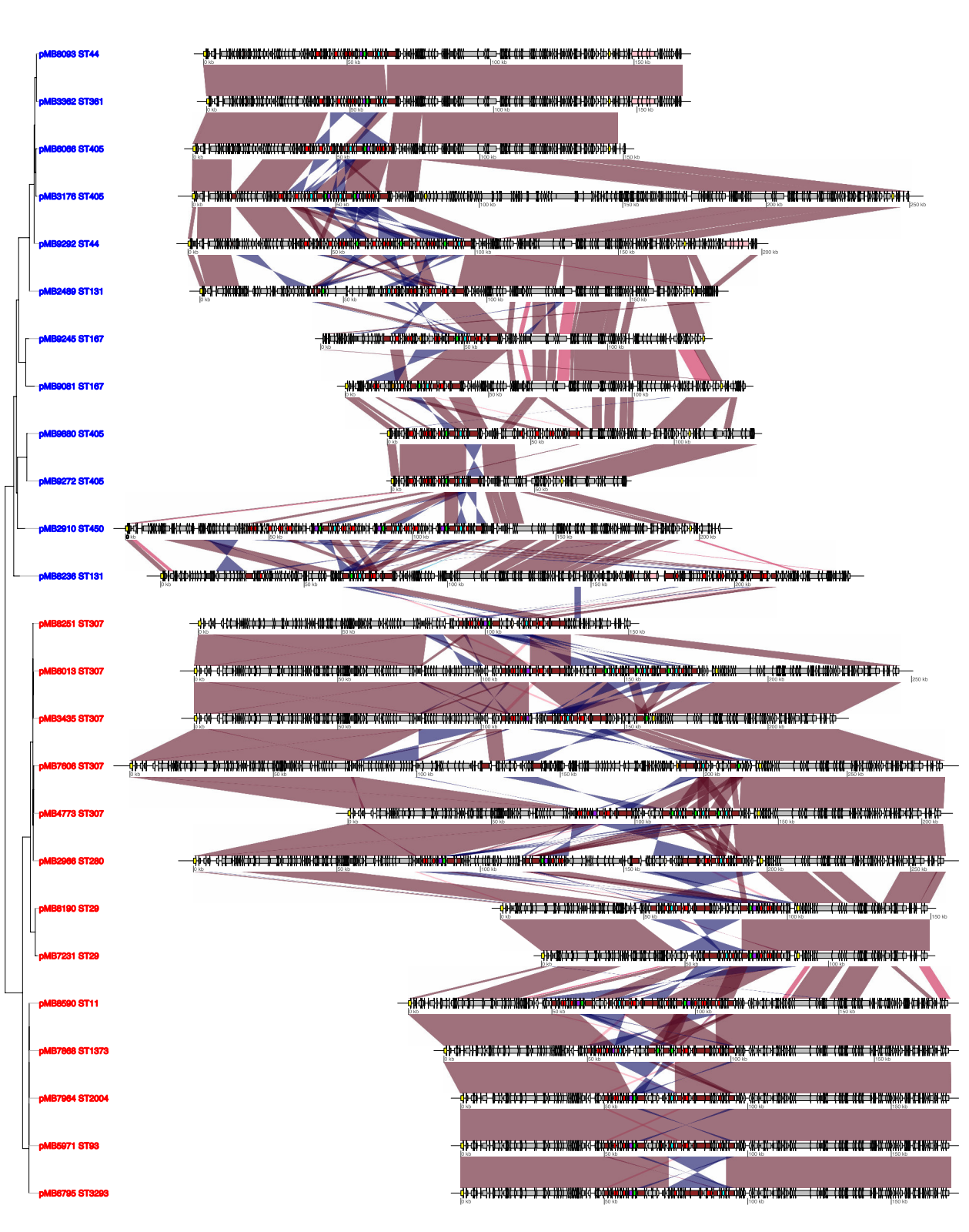

Supplement: FIG S3 [file msystems.00476-22-s0003.pdf]
